# A Stereological Study of the Toxic Effects of Cerium Oxide during Pregnancy on Kidney Tissues in Neonatal NMRI Mice

**DOI:** 10.1155/2020/9132724

**Published:** 2020-06-23

**Authors:** Afsaneh Nemati, Vahideh Assadollahi, Ilaria Peluso, Abolfazl Abbaszadeh, Mandana Beigi-boroujeni, Zahra Khanipur, Mohammadreza Gholami

**Affiliations:** ^1^Razi Herbal Medicines Research Center, Lorestan University of Medical Sciences, Khorramabad, Iran; ^2^Cancer and Immunology Research Center, Research Institute for Health Development, Kurdistan University of Medical Sciences, Sanandaj, Iran; ^3^Council for Agricultural Research and Economics, Research Center for Food and Nutrition (CREA-AN), Via Ardeatina 546, 00178 Rome, Italy; ^4^Hazrat Fatemeh Hospital, School of Medicine, Burn Research Center, Iran University of Medical Sciences, Tehran, Iran; ^5^Department of Anatomy, Kermanshah University of Medical Sciences, Kermanshah, Iran

## Abstract

**Background:**

Both antioxidant and prooxidant activities have been previously reported for cerium oxide (CeO_2_). The aim of this study was to investigate the effects of CeO_2_ at different doses on changes in kidney tissues and markers in neonatal mice.

**Methods:**

We randomly divided 30 pregnant NMRI mice into five groups (*n* = 6 per group)—a control group and four groups treated with intraperitoneal (i.p.) administration of different doses of CeO_2_ (10, 25, 80, or 250 mg/kg body weight (bw)) on gestation days (GD) 7 and GD14. At the end of the treatment period, we analyzed the kidney tissues and serum samples. The levels of two serum redox markers, malondialdehyde (MDA) and ferric reducing/antioxidant power (FRAP), were determined. Data were analyzed using one-way ANOVA and Tukey's test, and a *P* value of <0.05 was considered significant.

**Results:**

The mean total volumes of the renal corpuscle, glomeruli, and Bowman's capsule membranes significantly increased, and there was a significant decrease in the mean total volume of Bowman's space in the high-dose CeO_2_ group compared to that in the control group. No statistically significant differences existed in the serum levels of MDA and FRAP in the treated and control groups.

**Conclusion:**

Our results suggest that high doses of CeO_2_ impair fetal renal development in pregnant mice, which results in kidney damage. Therefore, CeO_2_ administration during pregnancy could have dose-dependent adverse effects on the developing kidneys in neonates.

## 1. Introduction

Cerium is the most abundant rare-earth metal and most active element in the lanthanide group. Cerium is a soft, ductile, and malleable metal with a color that ranges from iron-gray (commercial grade) to silver (pure form). Cerium compounds have the highest environmental activity compared to other members of the lanthanide group [1].

Cerium oxide (CeO_2_) is the most commonly used commercial compound of cerium [2]. Cerium oxide lanthanides are widely used as catalysts, oxygen sensors, in the manufacture of solar/fuel cells, and polishing agents in various fields [3–6]. The unique properties of CeO_2_, especially its low toxicity and high reducibility, have increased the use of micro- and nanosized CeO_2_ in various medical fields and led to significant advances in these fields [1]. The medical applications of CeO_2_ are due to its antioxidant, anti-inflammatory, and antibacterial properties and its high angiogenic potential. Cerium oxide is used to assist with the healing of various tissues such as the bones, skin, cardiac, and nerves. Recently, the transfer of drugs and genes by CeO_2_ nanoparticles and the use of CeO_2_ as treatments for cancer and other diseases has received much attention [7, 8].

Cerium, itself, has no properties and is not physiologically important for living organisms; however, soluble Ce^3+^ salts (sulfate, nitrate, chloride, phosphate, and hydroxide) contain various properties that are of medical importance. Cerium oxide is a pale yellow-white powder with the chemical formula CeO_2_ [9]. The autoregenerative cycle nature of CeO_2_ is due to the presence of an enormous number of surface defects and its ability to switch between Ce^3+^ and Ce^4+^ oxidation states. The formation of an oxygen vacancy in CeO_2_ is associated with reduced Ce^4+^ and Ce^3+^ oxidation. This property allows Ce to absorb or give off an electron from the active oxygen species, making them inactive and neutral, and indicates a key role in the ratio of the Ce^3+^/Ce^4+^ oxide in the antioxidant activity of CeO_2_ [9–11]. Cerium oxide is believed to function as a superoxide dismutase (SOD)/catalase mimetic [9, 12–15]. In an experiment on mice, the antioxidant properties of CeO_2_ nanoparticles inhibited active oxygen species and its potential for the treatment of oxidative stress was reported [16]. Oxidative stress is an imbalance between reactive oxygen species (ROS) and antioxidants in the body [17]. Researchers propose that CeO_2_ could be used to treat diseases associated with oxidative stress and inflammation [9, 10, 18, 19]. This ability of the nanoscale to neutralize ROS from a pool of high concentration polymer ligands suggests that nanoscale activity may not decrease in the physiological environment, even when coated with a protein corona. A study on the distribution of inhaled CeO_2_ nanoparticles in mice showed that the cells phagocytosed the nanoparticles [20, 21].

Previous studies on the effects of CeO_2_ in living organisms reported contradictory results. Some researchers reported that CeO_2_ caused oxidative stress in mitochondria and hepatocellular damage [22], inflammation in tissues such as the kidneys and liver [23], and DNA damage in peripheral blood leukocytes (PBL) and liver cells [24]. Cerium oxide can also cause lung fibrosis [25] and angiogenesis [26].

In contrast, other researchers reported that CeO_2_ could act as an antioxidant and be used for cancer prevention and treatment [27, 28]. In another study, the optimum concentration (10^−3^–10^−9^ M) of CeO_2_ increased cell division of primary fetal fibroblasts *in vitro* [29]. The results of a study showed that CeO_2_ nanoparticles reduced oxidative stress and inflammation in mice treated with diethylnitrosamine [30]. The protective effect of CeO_2_ nanoparticles in preventing tissue damage and oxidative stress induced by diabetes in pregnant mice has been reported [31]. Existing synthetic protocols have the ability to obtain CeO_2_ nanoparticles with different physical and chemical properties (shape, size, zeta potential, and cerium valence state). The synthesis method directly affects their biological activity [32]. The impact of these characteristics on toxicity, especially fetal toxicity, has not been elucidated.

The impact of a wide range of cerium nanoparticles stabilized by citrate on the growth of two-cell embryos was investigated. The results showed that the cerium nanoparticle concentrations had no toxic effects on fetal development [33].

Cerium oxide can cross the placenta and make its way to the liver, spleen, and lung tissues of adult, neonatal, and fetal mice, inducing tissue destruction and necrosis [34]. In addition, the results from our previous study have shown that high-dose CeO_2_ can have a devastating effect on testicular tissue development in neonatal mice [35].

The kidneys play a key role in regulating the body's homeostasis and excreting waste products [36]. Metanephric development begins in humans during week five of gestation and in mice at embryonic day (E) 10.5 [37]. There is an enhanced chance for exposure to CeO_2_ because of the increase in its various uses in daily life. Pregnant women are exposed to CeO_2_ via the skin, inhalation, foods, and medicines.

Congenital anomalies of the kidneys are among the most important anomalies [38]. When pregnant mice are exposed to CeO_2_, these particles can cross the placenta and accumulate in the fetal organs [22–24, 34]. CeO_2_ may hinder embryonic development and may have possible demographic impacts [34]. Given the importance of kidney development during pregnancy and the postpartum period, the present study is aimed at comparing histological changes in neonatal kidneys after their mothers were exposed to different doses of a CeO_2_ suspension during the gestational day (GD) 7 and GD14 of pregnancy.

## 2. Materials and Methods

### 2.1. Materials

Cerium (IV) oxide (CeO_2_) powder that had a diameter < 5 *μ*m, assay of 99.9% trace metal basis, and density of 7.13 g/ml at 25°C (lit.) was purchased from Sigma-Aldrich Corporation (St. Louis, MO, USA). We prepared the different doses of CeO_2_ in double-distilled (dd) water. Ultrasonic vibration (100 W, 30 kHz) was performed for 15 min before administration.

In this study, the selected doses were based on previous studies and doses lower than the lethal dose; 50% (LD50) were used for the animals [34, 39]. Based on the contradictory results of previous studies, we selected various doses that ranged from low to high to detect dose-dependent effects in the laboratory animals. We performed our experiment based on the characteristics reported by the manufacturer of CeO_2_ and previous experiments [1, 34, 40–42].

The different doses of CeO_2_ were prepared in double-distilled (dd) water. Ultrasonic vibration (100 W, 30 kHz) was performed for 15 min before administration.

Trichloroacetic acid ACS reagent, ≥99.0% (TCA); 2,4,6-tripyridyl-s-triazine (TPTZ); 2-thiobarbituric acid ≥ 98% (TBA); ferric chloride (FeCl_3_); sodium acetate; and hydrochloric acid-ACS reagent, 37% (HCl), were also purchased from Sigma-Aldrich Corporation.

### 2.2. Animals and Experimental Groups

We obtained adult NMRI mice (male : female ratio of 1 : 2) that had an average weight of 25–30 g from Pasteur Institute of Iran (Tehran, Iran). The animals were allowed to acclimate for one week under standard conditions that included a 12 : 12 h light/dark cycle with ad libitum access to food and water. Once acclimated to their new environment, the male and female mice were kept in a cage at a 1 : 2 ratio. The pregnant mice were placed in separate cages. The detection of a vaginal plug was considered to be gestation day (GD) 0. The pregnant mice were randomly divided into five groups (*n* = 6 per group): a control and four treatment groups. Mice in the treatment groups received intraperitoneal (i.p.) injections of different doses of CeO_2_ (10, 25, 80, or 250 mg/kg body weight (bw)) on GD7 and GD14.

In this experiment, 15-day-old neonates were used for histological evaluation of kidney tissues and serum biochemical parameters. Changes in body weights and kidney tissue in 2- and 6-day-old neonates were evaluated.

### 2.3. Histological Examinations of the Kidneys

The 15-day-old postpartum (dpp) offspring were weighed and anesthetized by chloroform. After dissection, blood samples were collected from the heart using a 1 cc syringe. The left kidneys from the mice were excised and rinsed with distilled water, weighed, and fixed for one week in a 10% formaldehyde solution. After tissue passage and paraffin block preparation, the paraffin blocks were sectioned into 5 *μ*m sections with a microtome and subsequently stained with Heidenhain's Azan stain [43]. We randomly selected nine sections from each kidney to evaluate the histological parameters.

### 2.4. Kidney Volume

We used the Cavalieri method to assess kidney volume [44]. First, we systematically selected 15 random tissue sections from all of the 5 *μ*m sections at the same interval. The predesigned point probe was randomly uniform on the image of each of the sections, and the points encountered with the whole kidney image were counted.

The kidney volume was calculated in all the slices by using the following formula:

(1) $$\begin{array}{c} V_{\left(\text{total}\right)}=\overset{n}{\underset{i=1}{\sum }}P\times a\left(p\right)\times t, \end{array}$$

where ∑_*i*=1_^*n*^*P* is the sum of the total points, “*t*” represents the thickness between selected sections, and “*a*(*p*)” is the level of the point probe.

Next, we calculated the cortex and medullary volumes. Tissues were chosen by regular, random sampling, and the average of 15 fields of view from each 5 *μ*m section was assessed at 100x magnification by placing the point probe on each field.

### 2.5. Volumes of the Cortex, Medulla, and Cortex Components

The total number of points that hit the probe with the entire field was ∑_*i*=1_^*n*^*P*_total_; the whole number of the points that hit the probe in the cortex was ∑_*i*=1_^*n*^*P*_cortex_ ; and the whole number of points that hit the probe in the medulla was ∑_*i*=1_^*n*^*P*_medulla_.

Volumetric density was calculated using the following formulas for the cortex and medulla:

(2) $$\begin{array}{c} V_{v\text{ cortex}}=\frac{\sum_{i=1}^{n}P_{\text{cortex}}}{\sum_{i=1}^{n}P_{\text{total}}},\\ V_{v\text{ medulla}}=\frac{\sum_{i=1}^{n}P_{\text{medulla}}}{\sum_{i=1}^{n}P_{\text{total}}}. \end{array}$$

We separately estimated the volumes of the cortex and medulla by multiplying the volume density of each by the kidney volume in each neonatal mouse.

(3) $$\begin{array}{c} V_{\text{cortex}}=Vv_{\text{cortex}}\times V_{\text{total}},\\ V_{\text{medulla}}=Vv_{\text{medulla}}\times V_{\text{total}}. \end{array}$$

We estimated the volume of the components of the cortex, proximal tubule (PT), and distal tubule (DT), with the lumen and their epithelium, glomeruli, and interstitial tissue by systematic random sampling. An average of 15 fields of view from each 5 *μ*m slide was assessed by placing a counting frame on each field. The total number of the points that hit the frame with the entire field of view was selected (∑_*i*=1_^*n*^*P*_total_), and the whole number of points that hit each component (∑_*i*=1_^*n*^*P*_*x*_) was shown. The volume density was calculated using the following formula:

(4) $$\begin{array}{c} V_{v\;x}=\frac{\sum_{i=1}^{n}P_{\left(x\right)}}{\sum_{i=1}^{n}P_{\text{total}}}, \end{array}$$

where “*x*” represents the PT and DT, lumen, epithelium, interstitial tissue, and glomeruli.

Then, using the following formula, we separately calculated the volumes of the PT and DT, lumen, epithelium, interstitial tissue, or glomeruli by multiplying the volume density of each in the cortical volume:

(5) $$\begin{array}{c} V_{x}=V_{\text{cortex}}\times V_{v\;x}. \end{array}$$

In the above formula, “*x*” represents the PT, DT, lumen, epithelium, interstitial tissue, and glomeruli.

### 2.6. Volume of Bowman's Capsule and Space

In order to obtain the volume of the glomeruli components, we first compared the whole number of the points that hit the frame with these components (∑_*i*=1_^*n*^*P*_*x*_) and the whole number of the points that hit the frame with each glomerulus (∑_*i*=1_^*n*^*P*_glomerulus_). The volumetric density of the glomerulus was calculated using the following formula:

(6) $$\begin{array}{c} V_{v\;x}=\frac{\sum_{i=1}^{n}P_{\left(x\right)}}{\sum_{i=1}^{n}P_{\text{glomerulus}}}, \end{array}$$

where “*x*” represents each of the components of the glomerulus (Bowman's capsule and space).

Then, the volume of Bowman's capsule and space were calculated by multiplying the volume density of each component in the volume of the glomerulus as follows:

(7) $$\begin{array}{c} V_{x}=V_{\text{glomerulus}}\times V_{vx}, \end{array}$$

where “*x*” represented any of the glomerulus components, namely, Bowman's capsule and space.

### 2.7. Length of Proximal Tubules (PT) and Distal Tubules (DT)

In order to calculate the length of the PT and DT from the 5 *μ*m slides of kidney tissue at 400x magnification, we used systematic random sampling to select 15 fields of view. The counting probe was randomly placed on each of the microscopic fields of view, and the number of tubules counted within the frame or those that collided with the reception lines was counted. The number of tubules that contacted the banned lines was not counted. Then, the longitudinal densities of the PT and DT were calculated from the following equation:

(8) $$\begin{array}{c} L_{V=}2\times \frac{\sum_{i=1}^{n}Q_{i}}{a/f\sum_{i=1}^{n}P_{i}}, \end{array}$$

where ∑*Q*_*i*_  is the sum of selected tubules, *a*/*f* is the desired frame level at the texture scale, and ∑*P*_*i*_ is the sum of the points of contact with the kidney tissue.

### 2.8. Serum Redox Markers

Blood samples were collected from 15-day-old neonatal hearts to estimate the serum redox markers, malondialdehyde (MDA) and ferric reducing/antioxidant power (FRAP).

### 2.9. Malondialdehyde (MDA) Levels

Buege and Aust's procedure was used to evaluate serum MDA levels. In this method, a solution that contained trichloroacetic acid (TCA; 15% g/ml), TBA (0.375%, g/ml), and hydrochloric acid (HCl, 25% normal) was prepared and the sera were combined in a 2 : 1 ratio and placed in a bain-marie for 15 min. The solution was placed in cold water and then centrifuged for 10 min. The absorbance of the solution was read using a spectrophotometer at a wavelength of 532 nm [45, 46].

### 2.10. Ferric Reducing/Antioxidant Power (FRAP) Assay

The FRAP assay was used to estimate the antioxidants. We combined 0.5 ml of serum with 1.5 ml of the reaction mixture. The degree of plasma regeneration is proportional to the concentration of this complex. At low pH, the reduction of the TPTZ-Fe^3+^ complex in the form of ferrous (Fe^2+^) creates a blue complex that has a maximum absorption of 593 nm. The degree of the regenerative capacity of the serum was measured by increasing the concentration of the above complex using a spectrophotometer. The FRAP assay directly evaluates the whole antioxidant power [47].

### 2.11. Statistical Analysis

Data were analyzed with SPSS 16 (Statistical Package for the Social Sciences), ANOVA, and Tukey's test. *P* values < 0.05 were considered statistically significant.

## 3. Results

### 3.1. Histological Evaluation of the Kidney Tissues

**Figure 1:**
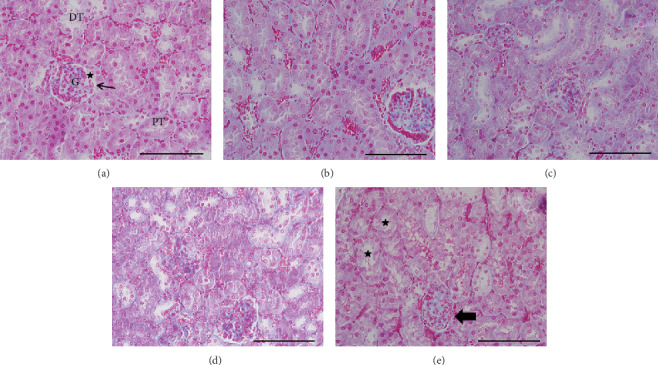
Microscopic images of kidney tissue from 15-day-old mice. The 5 *μ*m sections stained with Heidenhain's Azan show histopathologic changes in the kidney tissue. Magnification: 400x (scale bars =100 *μ*m). Control (a): renal tubules with a regular arrangement of epithelial cells and glomerulus with natural size components and structure (arrow: Bowman's capsule membrane; star: Bowman's capsule space). PT: proximal convoluted tubule; DT: distal convoluted tubule; G: glomerulus in the control group. Cerium oxide (CeO_2_); 10 mg/kg body weight (bw) (b), CeO_2_; 25 mg/kg bw (c): renal tubules with a regular arrangement of epithelial cells and glomeruli with natural size components and structure in the groups treated with 10 and 25 mg/kg bw CeO_2_. CeO_2_; 80 mg/kg bw (d): histological changes are not significant compared to the control group. CeO_2_; 250 mg/kg bw (e): vacuolization in the renal tubules, along with disruption, injury, and degeneration in PTs, vascularization in the interstitial kidney tissue, hypertrophy in the glomerulus, and reduced Bowman's capsule space in the 250 mg/kg bw CeO_2_ treatment group.

In the control group, we observed that the kidney tissues had a normal structure with regular tubules, cylindrical epithelial cells based on the basement membrane, lumen space, and normal glomeruli. In the group that received less than 250 mg/kg bw CeO_2_, the glomeruli were inflamed, and there was a significant increase in the volumes of the glomeruli and the membrane of the Bowman's capsule, along with a significant decrease in volume of Bowman's capsule space compared to the control group (*P* < 0.02). There were no statistically significant differences in the other groups treated with CeO_2_ compared to the control group (Figure 1).

### 3.2. Body and Kidney Weights

There were no statistically significant differences in body and kidney weights in the treatment and control groups (Table 1).

### 3.3. Volume of the Kidney, Cortex, Medulla, and Cortex Components

A comparison of kidney volumes in the treatment and control groups showed a significant decrease in the group that received the 250 mg/kg bw CeO_2_ dose (*P* < 0.02) (Table 2).

The cortex volume was significantly reduced in the group that received 250 mg/kg bw CeO_2_ (*P* < 0.03) compared to that in the control group. There was no statistically significant difference in the other groups treated with CeO_2_ compared to that in the control group. We also observed no statistically significant difference in medulla volume in the treatment groups compared to that in the control group.

There were significant increases in volume in the interstitial tissue (*P* < 0.01), renal corpuscle (*P* < 0.02), glomerulus (*P* < 0.02), and Bowman's capsule (*P* < 0.02) tissues in the 250 mg/kg bw CeO_2_ group compared with those in the control group. The other treatment groups showed no significant volume changes in these tissues when compared with the control group (Table 3).

A significant decrease was observed in the volume of Bowman's space in the 250 mg/kg bw CeO_2_ (*P* < 0.05) group compared with that in the control group; however, the other treatment groups did not significantly differ with the control group (Table 3).

The volume of the PT and its epithelium (*P* < 0.04) and the PT lumen (*P* < 0.05) decreased significantly in the group that received 250 mg/kg bw CeO_2_ compared to that in the control group (*P* < 0.05).

There was no significant difference between the volume of the DT and the epithelium and its lumen in the group that received 250 mg/kg bw of CeO_2_ compared with that in the control group (Table 4).

### 3.4. Lengths of Proximal Tubules (PT) and Distal Tubules (DT)

There was no significant difference between the DT and PT lengths in the treatment groups compared to that in the control group (Table 5).

### 3.5. Biochemical Evaluations

**Figure 2:**
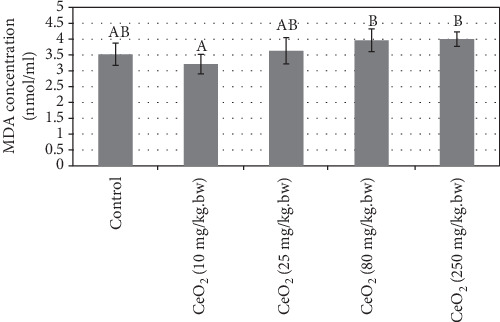
Comparison of the mean levels of serum malondialdehyde (MDA; nmol/ml) in the different groups of 15-day-old neonatal mice. Values are means ± SD. The means with different letter codes are significantly different from each other (ANOVA, Tukey's test, *P* < 0.05).

**Figure 3:**
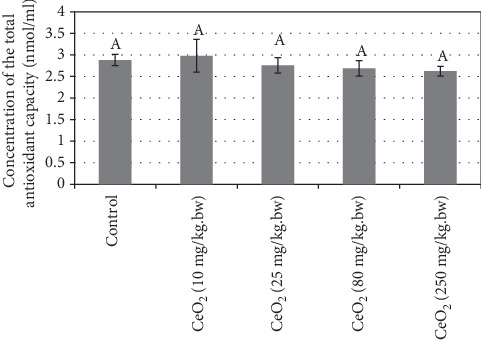
Comparison of the mean levels of serum total antioxidant capacity (TAC; nmol/ml) in the different groups of 15-day-old neonatal mice. Values are means ± SD. The means with different letter codes are significantly different from each other (ANOVA, Tukey's test, *P* < 0.05).

Statistical analysis of blood serum MDA showed no significant difference between treatment groups compared to the control group (Figure 2). In addition, statistical analysis of blood serum total antioxidant capacity (TAC) showed no significant difference between treatment groups compared to the control group (Figure 3).

## 4. Discussion

In this study, we administered i.p. injections of different doses of a CeO_2_ microparticle suspension to pregnant mice on GD7 and GD14 and examined their effects on neonatal mice kidney tissues by light microscopy. The selection of the CeO_2_ micropowder for this experiment was based on previous studies in which the toxic effects of CeO_2_ microparticles and their faster accumulation in the tissues of living organisms were confirmed [34, 48]. Exposure of pregnant mice to CeO_2_, according to the administered dose, caused changes in the neonatal kidney tissue.

Determining the average tissue and body weights is an important indicator for assessing the toxic effects of a substance on the body. In this study, body and kidney weight changes in offspring were measured at 2, 6, and 15 dpp. The results indicated that there were no significant differences in body and kidney weights between the experimental and control groups (Table 1).

Previous findings have indicated that CeO_2_ passes through the placenta [34]. In mice, the development of the metanephros is considered to begin at E10.5–11 and ends at 7–10 days after birth [49, 50]. The kidney, like all other major organs of the body, is susceptible to exposure to a wide range of chemicals during developmental periods. Regulated differentiation and proliferation of mesenchymal cells and urinary epidermal primordial cells cause nephrogenesis in the embryonic period [50]. Kidney development in mice is completed two weeks after birth [51]. Accordingly, we decided to investigate changes in kidney tissue 15 days after birth.

Studies have also shown that CeO_2_ can accumulate and cause inflammation in tissues such as the lungs, liver, and kidneys [23]. Cerium is capable of switching between the Ce^3+^ and Ce^4+^ states, which may aid the antioxidant property of CeO_2_. On the other hand, another investigation has shown that CeO_2_ causes ROS formation, inflammation, and DNA loss [19]. The result of one study indicated that CeO_2_ increased ROS formation and, consequently, induced oxidative damage in mitochondria [22].

Increased CeO_2_-induced ROS levels may be the cause of observed cellular damage and apoptosis. ROS production and oxidative stress might be due to the catalytic properties of CeO_2_, impaired mitochondrial function, or a combination of both mechanisms [24, 42].

ROS is capable of reacting with proteins, lipids, and nucleic acids, leading to lipid oxidation in biological membranes and the effects of enzymatic processes such as ion pump activity and DNA damage, thereby inhibiting transcription, repair, and apoptosis [52, 53]. As a result, lipid peroxidation destroys unsaturated fatty acids in the membranes [54]. This can be one reason for the decrease in cell volume and, ultimately, the decrease in kidney volume in the group that received 250 mg/kg bw CeO_2_ compared to that in the control group (Table 2).

Increased glomeruli volume, as representative of the renal and functional units of the kidney, can compensate for lost glomeruli function, adapt to new conditions, and remove toxins from the body [55, 56]. Glomeruli undergo hyperfiltration to control the conditions and maintain filtration, resulting in an increase in glomerular volume [57].

Oxidative stress contributes to kidney damage through several mechanisms. This primarily occurs through increased expression of the vascular endothelial growth factor (*VEGF*) gene in podocytes, endothelial cells, and renal mesangial cells that increase glomerular permeability and protein excretion through urine [58]. Growth factors increase the expression of collagen types I, III, IV, V, and VI, and the laminin and fibronectin proteins, which increases the extracellular matrix and thickening of the glomerular basement membrane [58, 59].

It has been shown that oxygen free radicals play a major role in inflammation in kidney interstitial tissue [60, 61]. Therefore, an increase in interstitial tissue volume in the group that received CeO_2_ at a dose of 250 mg/kg bw compared to that in the control group (Table 3) might indicate inflammation caused by CeO_2_.

In the present study, the lumen space of the PT in the group that received 250 mg/kg bw CeO_2_ was decreased compared to that in the control group (Table 4). This could be due to the destructive dose-dependent effect of CeO_2_ on tubules and the presence of necrosis, an apoptotic margin of the PT epithelial cells, and swelling of the epithelial cells of the wall of the tubule. It can be concluded that the swelling of PT wall cells reduces the lumen spaces of tubules [23].

The data of this study showed no statistically significant difference between serum MDA and FRAP levels in the treatment groups compared with the control group (Figure 3). Oxidative stress is due to reduced body resistance to oxidants and lower antioxidant levels in the blood. [22, 62]. According to other studies, antioxidant capacity *in vivo* depends on many factors such as environmental conditions (diet, etc.) [63, 64].

Studies of CeO_2_ in different animals showed that the level of CeO_2_ toxicity depended on the duration of exposure, tissue environment, and type of cell [19, 65].

Previous research has suggested that the effect and toxicity of CeO_2_ are closely related to the types of tissues and cells, as well as the type of animal and the duration of exposure [66–68].

Studies of the dose-dependent relationship of CeO_2_ effects on living organisms are complex. In one study, ICR mice were treated by oral gavage with one of three doses (10, 20, or 40 mg/kg bw/day) for six weeks. The accumulation of Ce particles in the nuclei of liver cells and mitochondria had a direct relationship to the increased dose [22]. The inflammatory effects of CeO_2_ nanoparticles were studied at different doses (2000, 3000, and 5000 mg/kg bw) administered daily for 14 days in CD-1 mice. The results did not show any relationship between the concentration used and toxic effects [23].

In the present study, animals exposed to the highest doses of CeO_2_ (250 mg/kg bw) showed significantly different histological parameters from their control counterparts. Animals exposed to the lowest doses (10, 25, and 80 mg/kg bw) did not show significant differences in histological parameters compared to the control group. Studies on the effect of CeO_2_ on living organisms *in vivo* and *in vitro* confirm our findings. Previous studies have shown that low-dose CeO_2_ can be used to treat cancer and eye diseases and is a powerful antioxidant [69, 70]. Therefore, according to review data, low-dose CeO_2_ may have beneficial and possibly protective effects.

According to histological data, the high dose of CeO_2_ in this experiment (250 mg/kg bw) was not tolerable for the animals. This dose could lead to toxic effects and oxidative stress, as well as disruption in the development of kidney tissues in mice. The present study results indicated that the dose of CeO_2_ could determine the presence of positive and negative effects from its various applications. However, additional research should be conducted to confirm these findings.

## 5. Conclusion

We observed significant increases in the mean total volume of the kidney, cortex, renal corpuscle, glomerulus, and membrane of Bowman's capsule and a significant decrease in the mean total volume of Bowman's space in the group that received 250 mg/kg bw of CeO_2_ compared to that in the control group. Our data showed no statistically significant differences between serum MDA and TAC levels in the treatment and control groups. According to our experiment, the efficacy of CeO_2_ on kidney development in neonatal mice was dose dependent. More studies should be conducted to investigate CeO_2_-induced renal damage in offspring exposed to CeO_2_ in utero.

## Figures and Tables

**Table 1:** Comparison of body weight (bw) and kidney weight in the study groups.

Group	bw (D_2_)	bw (D_6_)	bw (D_15_)	Kidney W (D_2_)	Kidney W (D_6_)	Kidney W (D_15_)
Control	1.87 ± 0.18^ab^	3.77 ± 0.18^ab^	7.13 ± 0.87^a^	0.015 ± 0.001^ab^	0.029 ± 0.002^ab^	0.042 ± 0.002^ab^
CeO_2_ (10 mg/kg bw)	1.86 ± 0.24^ab^	3.94 ± 0.34^a^	7.54 ± 0.8^8a^	0.016 ± 0.002^a^	0.030 ± 0.003^a^	0.044 ± 0.002^a^
CeO_2_ (25 mg/kg bw)	1.96 ± 0.11^b^	3.59 ± 0.2^ab^	6.87 ± 0.38^a^	0.013 ± 0.002^ab^	0.027 ± 0.003^ab^	0.040 ± 0.004^ab^
CeO_2_ (80 mg/kg bw)	1.76 ± 0.12^ab^	3.64 ± 0.27^ab^	6.82 ± 0.31^a^	0.013 ± 0.003^ab^	0.026 ± 0.002^ab^	0.038 ± 0.003^b^
CeO_2_ (250 mg/kg bw)	1.68 ± 0.09^b^	3.45 ± 0.2^b^	6.68 ± 0.47^a^	0.011 ± 0.001^b^	0.025 ± 0.001^b^	0.037 ± 0.002^b^

bw (D_2_): 2-day-old newborn body weight; bw (D_6_): 6-day-old newborn body weight; bw (D_15_): 15-day-old newborn body weight. Kidney W (D_2_): 2-day-old newborn kidney weight; Kidney W (D_6_): 6-day-old newborn kidney; Kidney W (D_15_): 15-day-old newborn kidney. Values are means ± SD. The means with different letter codes are significantly different from each other (ANOVA, Tukey's test, *P* < 0.05).

**Table 2:** Comparison of total kidney volume, cortex, and medulla in the study groups.

Group	Kidney V (mm^3^)	Cortex V (mm^3^)	Medulla (mm^3^)
Control	106 ± 8.21^ab^	86.4 ± 7.43^ab^	19.6 ± 1.14^ab^
CeO_2_ (10 mg/kg bw)	111.17 ± 10.14^a^	91.16 ± 8.84^a^	20 ± 1.78^a^
CeO_2_ (25 mg/kg bw)	102.5 ± 2.51^abc^	83.83 ± 2.13^abc^	19 ± 1.09^ab^
CeO_2_ (80 mg/kg bw)	99.17 ± 2.22^bc^	80.50 ± 2.16^bc^	18.66 ± 0.81^ab^
CeO_2_ (250 mg/kg bw)	93.67 ± 3.44^c^	75.83 ± 3.6^c^	17.83 ± 0.75^b^

Values are means ± SD. The means with different letter codes are significantly different from each other (ANOVA, Tukey's test, and *P* < 0.05).

**Table 3:** Comparison of interstitial tissue, glomerulus, and Bowman's capsule and space volumes in the study groups.

Group	InT (mm^3^)	Renal corpuscle (mm^3^)	Glomerulus (mm^3^)	Bowman's capsule (mm^3^)	Bowman's space (mm^3^)
Control	6.25 ± 0.42^a^	4.12 ± 0.35^a^	2.49 ± 0.46^a^	0.65 ± 0.12^ab^	0.97 ± 0.04^a^
CeO_2_ (10 mg/kg bw)	6.31 ± 1.08^a^	4.04 ± 0.5^a^	2.49 ± 0.56^a^	0.51 ± 0.11^a^	1.03 ± 0.12^a^
CeO_2_ (25 mg/kg bw)	6.23 ± 1.11^a^	4.13 ± 0.36^a^	2.62 ± 0.38^ab^	0.60 ± 0.05^a^	0.89 ± 0.11^ab^
CeO_2_ (80 mg/kg bw)	7.29 ± 0.82^ab^	4.33 ± 0.56^ab^	2.64 ± 0.58^ab^	0.81 ± 0.8^bc^	0.87 ± 0.10^ab^
CeO_2_ (250 mg/kg bw)	8.03 ± 0.40^b^	5.06 ± 0.49^b^	3.47 ± 0.47^b^	0.84 ± 0.08^c^	0.74 ± 0.07^b^

Values are means ± SD. The means with different letter codes are significantly different from each other (ANOVA, Tukey's test, *P* < 0.05).

**Table 4:** Comparison of the volumes of the renal structures in the study groups.

Group	PT (mm^3^)	PT (E) (mm^3^)	PT (L) (mm^3^)	DT (mm^3^)	DT (E) (mm^3^)	DT (L) (mm^3^)
Control	64 ± 5.33^ab^	48 ± 4^ab^	16 ± 1.33^a^	17.2 ± 0.83^ab^	11.62 ± 0.91^ab^	5.57 ± 0.38^b^
CeO_2_ (10 mg/kg bw)	68.16 ± 7.13^a^	51.12 ± 5.35^a^	17.04 ± 1.78^a^	19 ± 2.19^a^	12.39 ± 1.12^a^	6.61 ± 1.12^a^
CeO_2_ (25 mg/kg bw)	62.16 ± 1.94^abc^	46 ± 45 ± 1.39^abc^	15.54 ± 0.48^ab^	17.33 ± 0.51^ab^	11.7 ± 0.32^ab^	5.63 ± 0.36^ab^
CeO_2_ (80 mg/kg bw)	59.16 ± 1.32^bc^	44.37 ± 0.99^bc^	15.12 ± 0.73^ab^	16.5 ± 0.54^b^	11.19 ± 0.51^ab^	5.31 ± 0.26^b^
CeO_2_ (250 mg/kg bw)	56.08 ± 3.47^c^	42.08 ± 2.59^c^	14 ± 0.88^b^	15.75 ± 0.75^b^	10.53 ± 0.55^b^	5.21 ± 0.24^b^

PT: proximal convoluted tubule; DT: distal convoluted tubule; lumen: L; epithelium: E. Values are means ± SD. The means with different letter codes are significantly different from each other (ANOVA, Tukey's test, *P* < 0.05).

**Table 5:** Lengths of the proximal tubules (PT) and distal tubules (DT) in the study groups.

Group	PT (m)	DT (m)
Control	37.81 ± 1.59^a^	22.46 ± 4.86^a^
CeO_2_ (10 mg/kg bw)	38.69 ± 1.22^a^	25.33 ± 1.3^a^
CeO_2_ (25 mg/kg bw)	37.47 ± 1.19^a^	25.26 ± 1.12^a^
CeO_2_ (80 mg/kg bw)	35.53 ± 3.65^a^	22.70 ± 3.9^a^
CeO_2_ (250 mg/kg bw)	35.04 ± 3.81^a^	19.92 ± 5.21^a^

Values are means ± SD. The means with different letter codes are significantly different from each other (ANOVA, Tukey's test, *P* < 0.05).

## Data Availability

No data were used to support this study.

## Abbreviation

Bw: Body weight
Ce: Cerium
CeO_2_: Cerium (IV) oxide
CTGF: Connective tissue growth factor
DD: Double distilled
DPP: Days postpartum
D2: 2-day-old infant
D6: 6-day-old infant
DT: Distal tubules
°C: Degree centigrade
Fig: Figure
g: Gram
GD: Gestational day
G: Glomerulus
HCL: Hydrochloric acid
H: Hour
i.p.: Intraperitoneal
*μ*L: Microliter
MDA: Malondialdehyde concentration
*μ*m: Micrometer
min: Minute
mm^3^: Cubic millimeters
mg/kg bw: Milligrams per kilogram of body weight
ml: Milliliter
Nm: Nanometer
Nmol: Nanomolar
PBL: Peripheral blood leukocytes
PT: Proximal tubules
PDGF: Platelet-derived growth factor
KCL: Potassium chloride
LDH: Lactate dehydrogenase
REEs: Rare earth elements
TAC: Total antioxidant capacity
TCA: Trichloroacetic acid
TPTZ: 2,4,6-Tripyridyl-s-triazine
TBA: Thiobarbituric acid
TGF-1: Transforming growth factor-1
VEGF: Vascular endothelial growth factor
V: Volume
W: Weight.
